# Aquaporin-1 attenuates macrophage-mediated inflammatory responses by inhibiting p38 mitogen-activated protein kinase activation in lipopolysaccharide-induced acute kidney injury

**DOI:** 10.1007/s00011-019-01285-1

**Published:** 2019-09-16

**Authors:** Bohui Li, Chunmei Liu, Kaihong Tang, Xuening Dong, Longge Xue, Guangming Su, Wenzheng Zhang, Yingyu Jin

**Affiliations:** Department of Laboratory Diagnosis, The First Affiliated Hospital of Harbin Medical University, Harbin, 150001 Heilongjiang China

**Keywords:** Aquaporin-1, AKI, Macrophage polarisation, p38 MAP kinase, Nuclear factor-κB

## Abstract

**Objective:**

This study was designed to investigate the role of AQP1 in the development of LPS-induced AKI and its potential regulatory mechanisms in the inflammatory responses of macrophages.

**Methods:**

Male Wistar rats were injected intraperitoneally with LPS, and biochemical and histological renal damage was assessed. The levels of inflammatory mediators, macrophage markers and AQP1 in blood and kidney tissues were assessed by ELISA. RTPCR was used to assess changes in the relative levels of AQP1 mRNA induced by LPS. Western blot and immunofluorescence analyses were performed to assay the activation of the p38 MAPK and NF-κB pathways, respectively. The same detection methods were used in vitro to determine the regulatory mechanisms underlying AQP1 function.

**Results:**

AQP1 mRNA levels were dramatically decreased in AKI rats following the increased expression of inflammatory factors. In vitro experiments demonstrated that silencing the AQP1 gene increased inflammatory mediator secretion, altered the classical activation of macrophages, greatly enhanced the phosphorylation of p38 and accelerated the translocation of NF-κB. Furthermore, these results were blocked by doramapimod, a p38 inhibitor. Therefore, these effects were mediated by the increased phosphorylation of p38 MAPK.

**Conclusion:**

Our results suggest that altered AQP1 expression may be associated with the development of inflammation in AKI. AQP1 plays a protective role in modulating acute renal injury and can attenuate macrophage-mediated inflammatory responses by downregulating p38 MAPK activity in LPS-induced RAW264.7 cells. The pharmacological targeting of AQP1-mediated p38 MAPK signalling may provide a novel treatment approach for AKI.

## Introduction

Inflammation is a complex biological response to injury caused by noxious physical or chemical factors and plays an important role in pathogen removal and tissue repair after injury [1]. Acute kidney injury (AKI) is an abnormal inflammatory response in critically ill patients that is induced by bacterial, viral or fungal infection and is associated with high morbidity and mortality. Lipopolysaccharide (LPS)-induced AKI has been widely used as a model for the study of conditions such as renal endothelial dysfunction and renal inflammation [2]. Although scientists have discovered that innate immunity and inflammatory signalling pathways are involved in the pathogenesis of AKI, there is no effective therapy to treat or prevent AKI by modulating inflammation [3, 4].

Macrophages, a type of immune cell involved in the initiation, development, and resolution of inflammation, play significant roles in the development of renal injury. Due to differences in the immune microenvironment, they are activated and polarised into several phenotypes. To date, accumulating evidence has shown that after stimulation by pathogens or injuries, macrophages undergo differentiation into the M1 phenotype, which is a proinflammatory state that promotes the removal of infected or apoptotic cells. In contrast, anti-inflammatory M2 macrophages play an important role in tissue repair [5]. Macrophages may determine the outcomes of renal diseases. In vivo studies utilising animal models of infectious organ injury suggest that depleting macrophages or reducing macrophage activation can protect the kidney from pathogens or stimuli resulting from endogenous injury [6, 7]. Recently, the Acute Dialysis Quality Initiative (ADQI) XIII Work Group proposed that macrophages are a therapeutic target involved in the process of tissue repair after AKI [8].

Mitogen-activated protein kinases (MAPKs) play a vital role in the transduction of extracellular signals caused by extracellular stimuli. These proteins are involved in LPS-induced inducible NO synthase (iNOS) and cyclooxygenase 2 (COX-2) expression in macrophages [9]. p38 represents a MAPK signal transduction pathway and plays an essential role in macrophage-mediated inflammatory responses and diseases, including endotoxin-induced shock, diabetes and acute lung inflammation [10]. In response to inflammatory and stress stimuli, p38 is rapidly phosphorylated, and its expression is upregulated. Furthermore, some downstream transcription factors are activated, such as nuclear factor kappa B (NF-κB), which is a pivotal regulator of proinflammatory mediator gene expression and induces the transcription of proinflammatory molecules such as TNF-α, IL-1β, IL-6, iNOS, and COX-2 [11].

Aquaporins (AQPs) are a group of transmembrane channels that mainly regulate intracellular and intercellular water flow; 13 AQPs have been identified to date (AQP0–AQP12) and are widely distributed in various tissues and organs. AQPs are the main molecules that allow the kidneys to maintain a normal urine concentration and substance metabolism. In the uropoietic system, AQP1, the first water channel discovered, is expressed in the apical and basolateral plasma membrane of the proximal tubule and in the thin descending limbs of Henle to mediate water reabsorption [12]. AQP1 is a highly selective water-permeable channel and plays an essential role in countercurrent multiplication systems. In endotoxaemia-induced AKI, increased polyuria and more severe tubular injury were observed in AQP1-null mice compared with that in WT mice [13]. AQP1 may also serve as a sensitive biomarker because it plays an important role in inflammation. However, the role of AQP1 in the renal inflammatory immune response remains unclear. Our previous experiments have suggested that AQP1 plays anti-inflammatory and antiapoptotic functional roles, possibly via modulation of the p38 and ERK1/2 pathways. In this study, we used an LPS-induced AKI mouse model to further evaluate the functional mechanisms of AQP1 at different stages of AKI. Furthermore, we investigated whether AQP1 attenuates inflammatory responses via the p38 MAPK and NF-κB/IκBα signalling pathways in RAW264.7 macrophages to determine whether AQP1 is a potential therapeutic target for AKI.

## Materials and methods

### Experimental animals

Studies were performed on 8- to 10-week-old male Wistar rats initially weighing 180–220 g that were obtained from the Experimental Animal Center at the First Hospital of Harbin Medical University (China). The rats were maintained on a standard rodent diet and had free access to water. All rats (*n* = 24) were randomly assigned into one of the two experimental groups: the control group (*n* = 12), injected intraperitoneally with 600 μl of 0.9% saline, or the LPS group (*n* = 12), injected intraperitoneally with 10 mg/kg body weight LPS (*Escherichia coli* serotype 0111:B4, Sigma Aldrich, USA) in 600 μl of 0.9% saline. According to the time points after liquid injection (12, 24, 48 and 72 h), each group was further divided into four subgroups (*n* = 3/group). Both kidneys were removed, and the left kidney from each rat was kept at − 80 °C in a freezer, while the other kidney was fixed in 4% paraformaldehyde at 4 °C.

### Biochemical analysis

Rats were injected intraperitoneally with 10% chloral hydrate (3 ml/kg body weight), and blood samples were then collected from the heart and centrifuged at 3500 rpm for 5 min at room temperature; the supernatant was stored at − 80 °C. An automatic biochemical analyser (Vitros V5600, Johnson, USA) was used to determine the blood urea nitrogen (BUN) and serum creatinine levels to assess renal function.

### Histological analyses

The right kidneys were fixed in 4% paraformaldehyde and embedded in paraffin. All kidney tissue slides were stained with haematoxylin–eosin (H&E) and observed under a light microscope (Olympus CX43, Tokyo, Japan).

### Cell culture and transfection

RAW264.7 cells (ATCC^®^ TIB-71™) were obtained from the American Type Culture Collection and cultured at 37 °C in DMEM supplemented with 10% foetal bovine serum, 100 units/ml penicillin, and 100 μg/ml streptomycin in a humidified atmosphere containing 5% CO_2_.

RAW264.7 cells were transfected with 100 nM si-AQP1 (Guangzhou RiboBio Co., Ltd., Guangzhou, China) for 48 h using Lipofectamine^®^ 3000 (Invitrogen; Thermo Fisher Scientific, Waltham, USA) according to the manufacturer’s protocol. Following transfection, the cells were incubated with the p38 MAPK inhibitor doramapimod (TOCRIS, Turkey) at 100 nmol/l for 2 h and stimulated with 1 μg/ml LPS for 4, 24 and 48 h. The cell supernatants were collected for the enzyme-linked immunosorbent assay (ELISA), and the total RNA and protein prepared from the cells were used for reverse transcription-quantitative polymerase chain reaction and Western blot analyses.

### Enzyme-linked immunosorbent assay (ELISA)

The concentrations of four cytokines, tumour necrosis factor alpha (TNF-α), interleukin 6 (IL-6), iNOS, and Arg-1, and the concentration of AQP1 in plasma and kidney homogenates were detected with rat ELISA kits (Boster, Wuhan, China). The cytokine concentrations in cell supernatants were quantified using mouse ELISA kits. The procedures were performed according to the manufacturers’ instructions.

### RNA extraction and quantitative real-time PCR

Total RNA was extracted from mouse kidney tissues and RAW264.7 cells using TRIzol reagent (Axygen; Corning Incorporated, NY, USA) according to the manufacturer’s protocol. Purified RNA (1 µg/µl) was reverse-transcribed into cDNA using a Script cDNA Synthesis Kit (Bio-Rad Laboratories, Inc., CA, USA) at 37 °C for 15 min and at 98 °C for 5 min. RT-qPCR was carried out using a Bio-Rad CFX96 Optics Real-Time PCR System (Bio-Rad Laboratories, Inc., CA, USA). cDNA was amplified by polymerase chain reaction using specific primers (Table 1). The following PCR conditions were used for AQP1 and β-actin: 40 cycles of denaturation at 95 °C for 5 s, annealing at 60 °C for 10 s, and extension at 72 °C for 15 s. The AQP1 and β-actin primers produced amplified products of 129 and 70 bp, respectively.

**Table 1:** Primers used in the qPCR analysis

Primer	Sequence
AQP1	Forward primer 5′-CCTGCTGGCCATTGACTACA-3′
AQP1	Reverse primer 5′-TGGTTTGAGAAGTTGCGGGT-3′
β-Actin	Forward primer 5′-CGCGAGTACAACCTTCTTGC-3′
β-Actin	Reverse primer 5′-CGTCATCCATGGCGAACTGG -3′

### Immunofluorescence

Kidney tissue sections and cells were fixed in 4% paraformaldehyde for 15 min. After rinsing with PBS, the sections and coverslips with adherent cells were incubated in a goat anti-rabbit activated NF-κB monoclonal antibody (dilution 1:500) (Abcam, London, UK). Subsequently, they were incubated for 1 h with FITC-conjugated goat anti-rabbit IgG at room temperature. All images were captured with a confocal microscope (Olympus Fluoview1000, Tokyo, Japan).

### Western blot analyses

The concentrations of proteins obtained from kidney tissues and RAW264.7 cells were determined using an Enhanced BCA Protein Assay Kit (Beyotime, Shanghai, China). Equal amounts of protein (50 μg) were separated by 10% SDS-PAGE and transferred onto PVDF membranes (Millipore, MA, USA) using a wet transfer apparatus.

The membranes were blocked with 5% nonfat dry milk in TBST for 1 h and then incubated with primary antibodies against phosphorylated p38 (Beverly, MA, USA) at 4 °C overnight.

The blots were washed four times with Tween 20/Tris-buffered saline (TBST) and incubated with a secondary HRP-conjugated antibody for 2 h at room temperature. Then, the blots were visualised with chemiluminescence reagents. The densities of the bands were measured with Pierce ECL Western Blotting Substrate.

### Statistical analysis

Statistical analyses were carried out using GraphPad Prism 5.0 software (La Jolla, CA, USA). The data are expressed as the mean ± standard error of the mean (SEM). The treatment effects were analysed using ANOVA and two-tailed Student’s *t* tests. The statistical significance was set at *p < 0.05, **p < 0.01, or ***p < 0.001.

## Results

### Changes in renal function and inflammatory mediators in rats at different stages of endotoxaemic acute kidney injury

**Fig. 1:**
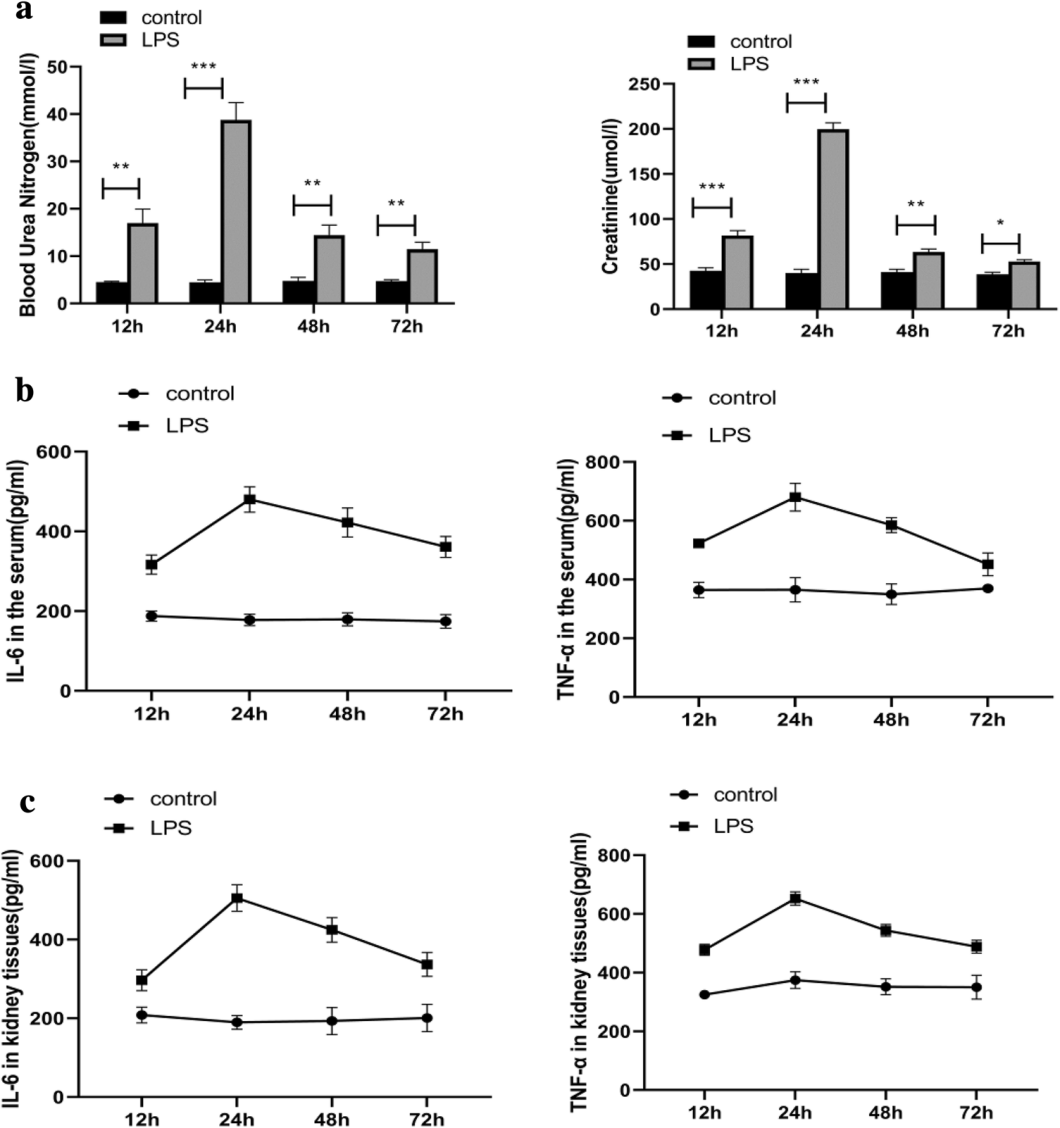
Inflammatory reaction in blood and kidney tissues resulting from endotoxaemic acute kidney injury in rats. **a** Serum BUN and creatinine levels were measured to assess renal function. **b** The concentrations of IL-6 and TNFα in the serum of rats from different groups were measured by ELISA. **c** The concentrations of IL-6 and TNFα in kidney tissues from different groups were measured by ELISA. *TNF-α* tumour necrosis factor-α, *IL-6* interleukin-6

**Fig. 2:**
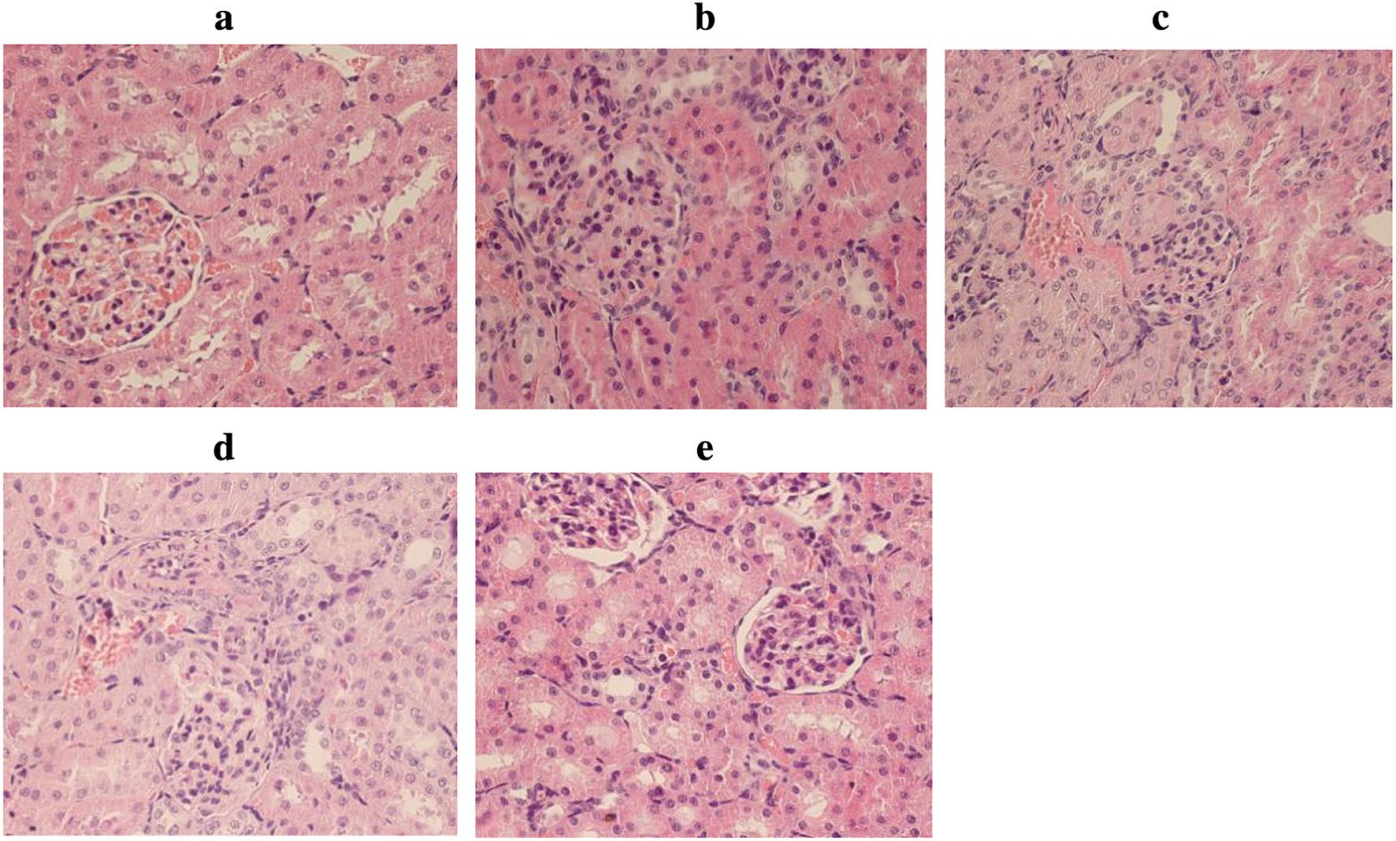
Morphological examination of kidney tissues. The kidney tissues of rats were prepared for histological analysis after LPS treatment and stained with haematoxylin (H&E staining; original magnification 400×). **a** Control group: normal kidney tissue; **b** LPS 12 h group; **c** LPS 24 h group; **d** LPS 48 h group; **e** LPS 72 h group

The LPS groups exhibited significant increases in BUN and Scr (Fig. 1a) levels at various time points (12, 24, 48, and 72 h) compared with those in the control group. As determined by ELISA, the levels of the proinflammatory mediators IL-6 and TNF-α (Fig. 1b) in blood were significantly increased at 12 h after LPS administration, peaked at 24 h, and then dropped at 48 h. This trend indicates that renal injury tends to recover at 48 h. At the same time, the changes in the IL-6 and TNF-α (Fig. 1c) concentrations in kidney tissues were consistent with the serum results. In addition, H&E staining followed by light microscopy showed that the kidney tissue in control rats was not histologically altered (Fig. 2a). Oedema in renal tubular epithelial cells and inflammatory cell infiltration were observed at 12 h after LPS treatment (Fig. 2b) and were time dependent to a certain degree. Compared with that at the 24-h time point (Fig. 2c), infiltration was decreased slightly at 48 h (Fig. 2d) and 72 h (Fig. 2e) after LPS treatment.

### Macrophage phenotype transition during endotoxaemic acute kidney injury

**Fig. 3:**
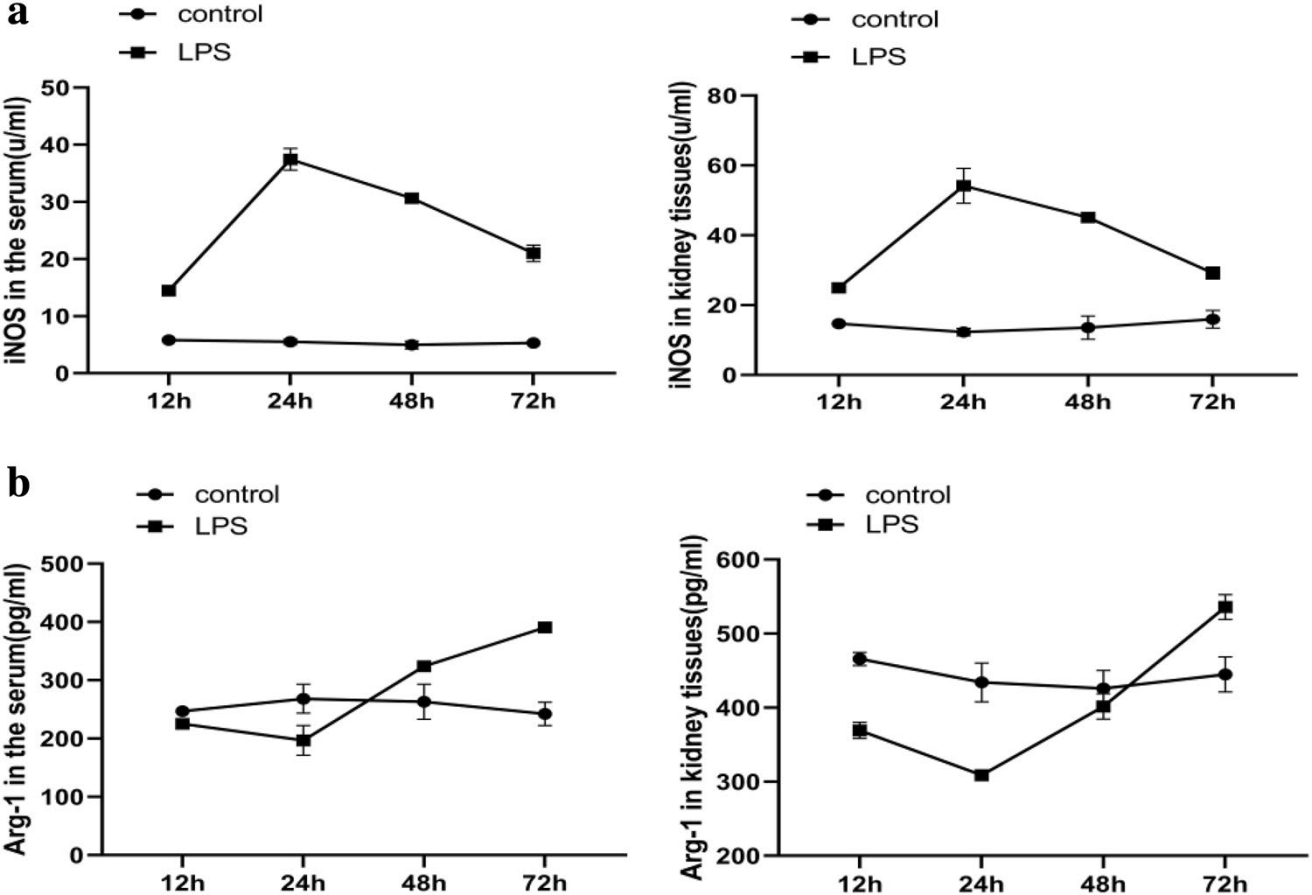
Changes in macrophage phenotypes in AKI rats. **a** The expression levels of the M1 macrophage cytokine iNOS in serum and kidney tissues. **b** The expression levels of the M2 macrophage cytokine Arg-1 in serum and kidney tissues. *iNOS* inducible nitric oxide synthase, *Arg-1* arginase 1

We used the ELISA method to determine the expression levels of M1/M2 macrophage markers at different time points in rats. Compared to those in control rats, high levels of the M1 macrophage marker iNOS were detected at 12 h and 24 h after injury in LPS rats, which was followed by a subsequent decrease (Fig. 3a). In contrast, the expression level of the M2-dependent cytokine Arg-1 was lowest at 24 h and was slightly increased at 48 h (Fig. 3b). These results indicate that the macrophages underwent M2 differentiation at 48 h, and the expression of M2 macrophage markers was obvious at 72 h after LPS-induced AKI. Moreover, persistent proinflammatory macrophage expression was associated with kidney injury.

### Changes in AQP1 protein and mRNA expression levels in rats at different stages of endotoxaemic acute kidney injury

**Fig. 4:**
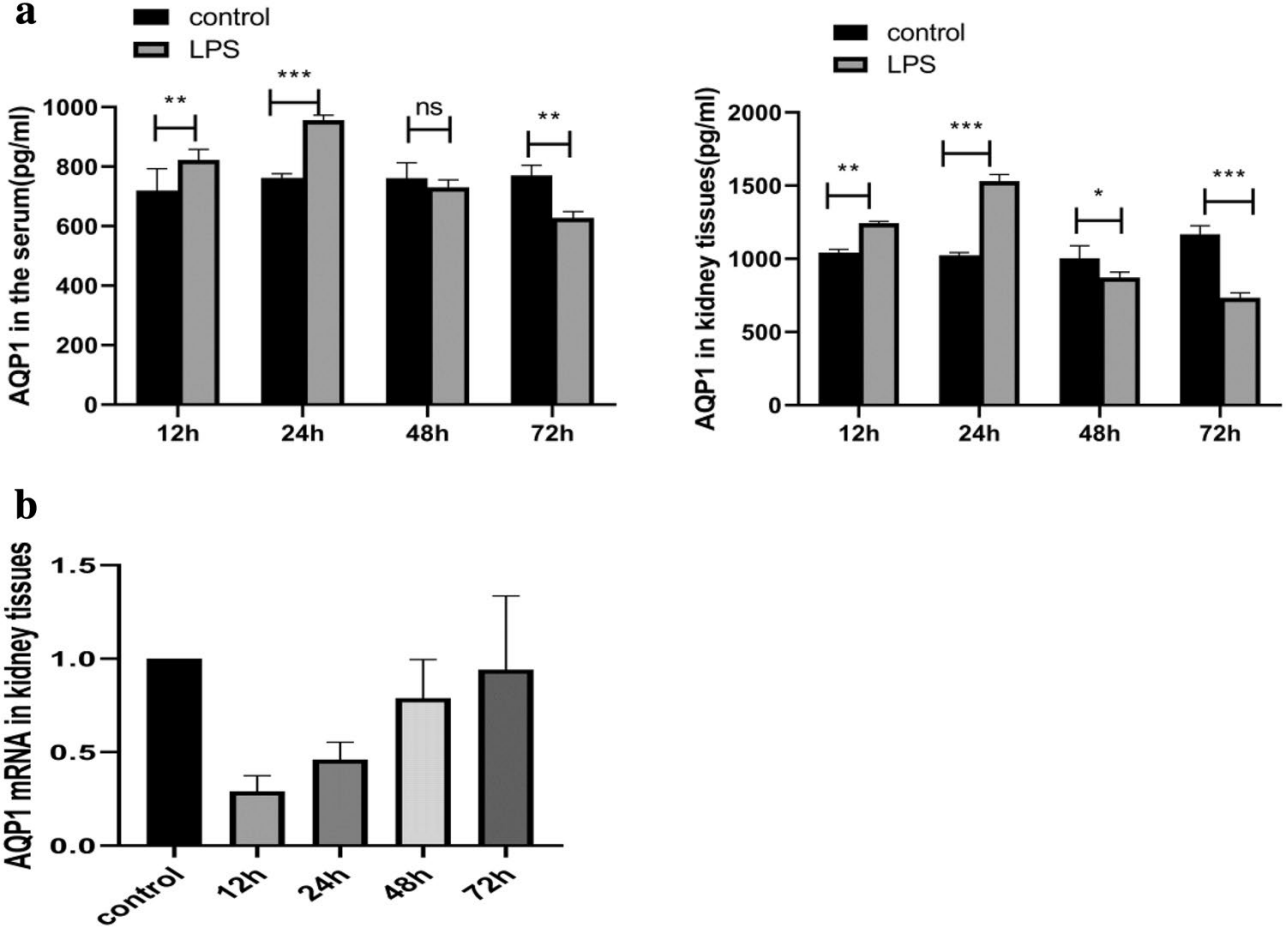
AQP1 expression in blood and kidney tissues in rats with endotoxaemic acute kidney injury. **a** The level of AQP1 expression in blood and kidney tissues. **b** AQP1 mRNA expression in kidney tissues from different groups. The results are expressed as the percentage of mRNA relative to that in control cells. The data represent the mean of three independent experiments

To determine the changes in AQP1 during the development of AKI, the AQP1 levels in plasma and kidney homogenates were detected by ELISA (Fig. 4a). The level of AQP1 was increased at 12 h, and it was significantly increased at 24 h compared with that in control rats and then subsequently returned to physiological levels. qRT-PCR analysis was used to determine the level of AQP1 mRNA in rat kidney tissues (Fig. 4b), revealing significantly inhibited expression at 12 h after treatment with LPS and a 3.44-fold decrease compared to the levels in control rats. During the subsequent hours, the AQP1 mRNA expression level showed a gradually increasing trend, but the expression level was always lower than that in the control group.

### The p38 MAPK pathway was activated following endotoxaemic-induced AKI

**Fig. 5:**
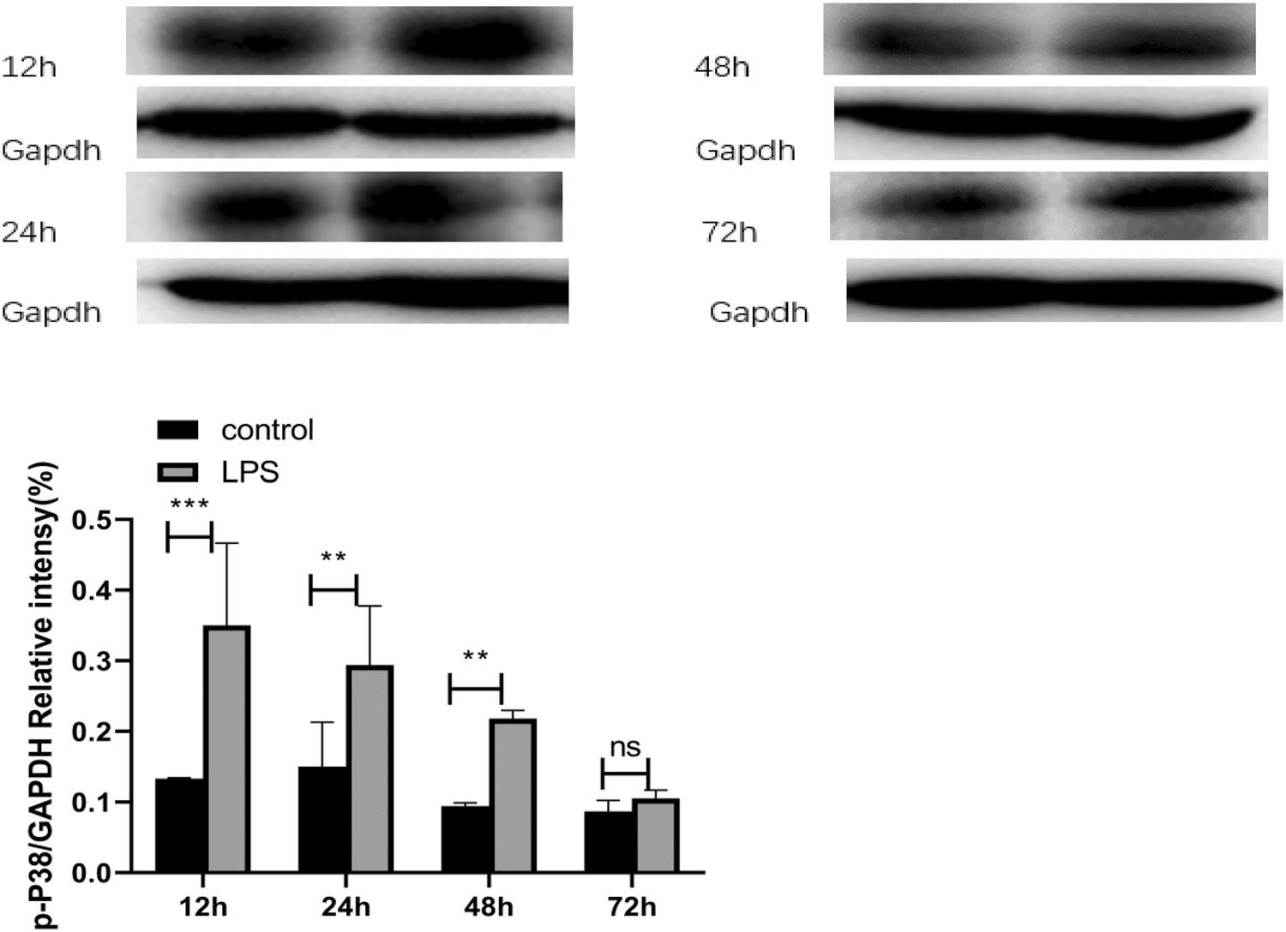
The activation of inflammatory pathways in LPS-induced AKI. **a** The phospho-p38 levels were determined by Western blot analysis. GAPDH was used as an internal control

Western blot assays indicated that LPS significantly induced the phosphorylation of p38 at 12 and 24 h (Fig. 5a).

### Gene silencing of AQP1 promoted the activation of p38 mitogen-activated protein kinase (MAPK) in LPS-stimulated RAW264.7 cells

**Fig. 6:**
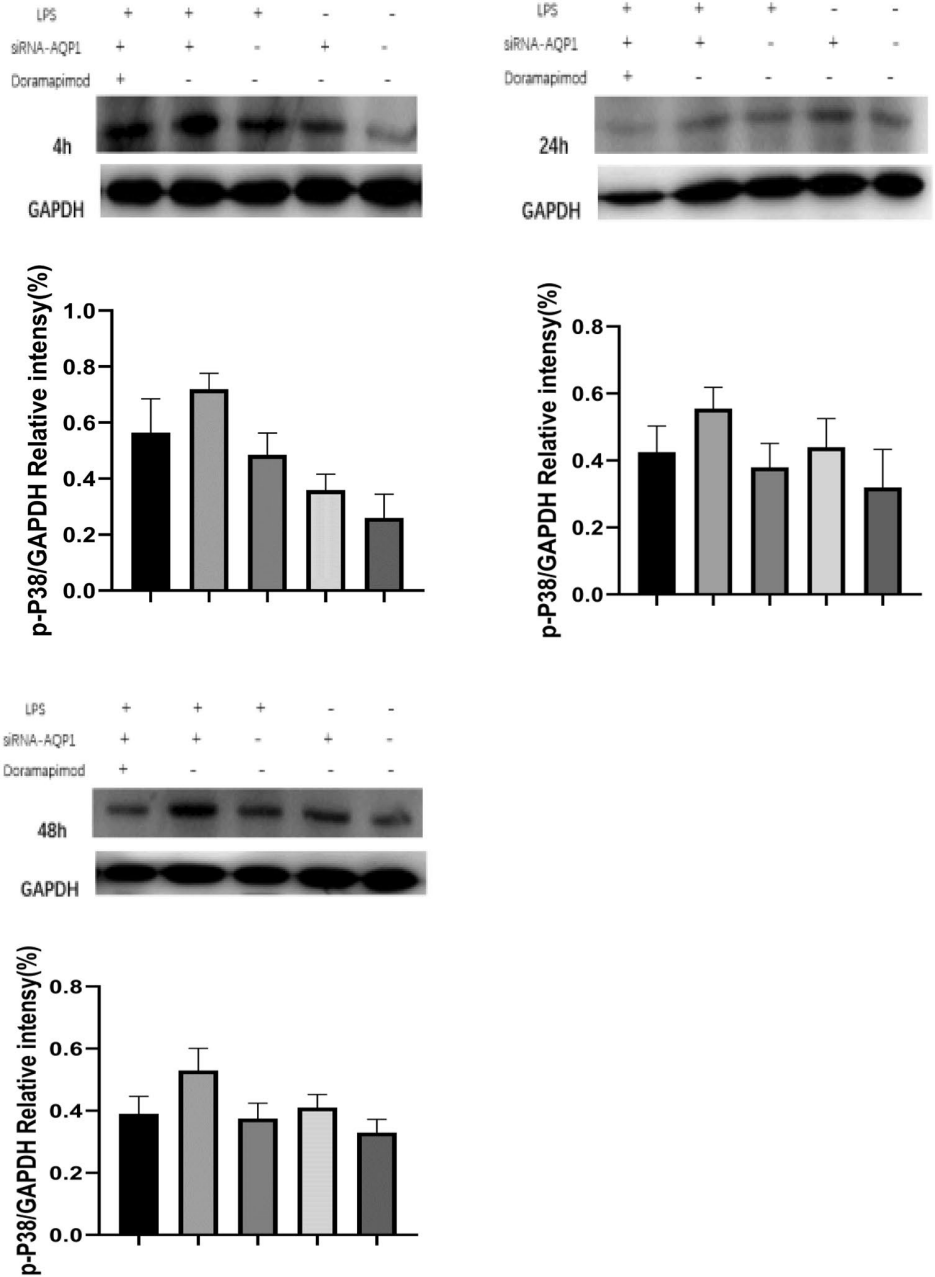
Effects of AQP1 on LPS-activated p38 MAPK signalling. Following transfection with siRNA-AQP1, RAW264.7 cells were treated with 1 μg/ml LPS for 4, 24 and 48 h (**a**–**c**). The phospho-p38 protein levels were measured by Western blot. Blots are representative of three independent experiments. Control: cells incubated with the vehicle alone. LPS group: cells incubated with only 1 μg/ml LPS. siRNA-AQP1: cells transfected with siRNA-AQP1 alone. siRNA-AQP1 + LPS: cells transfected with siRNA-AQP1 and then incubated with 1 μg/ml LPS. siRNA-AQP1 + Doramapimod + LPS: cells transfected with siRNA-AQP1, incubated with doramapimod for 2 h and stimulated with 1 μg/ml LPS. Doramapimod (RIRB796): inhibitor of the p38 pathway

To explore the possible mechanisms involved in AQP1-mediated inflammatory protection in AKI, LPS-induced inflammatory responses were studied in cultured RAW264.7 cells that were transfected with si-AQP1. p38 phosphorylation was analysed by Western blot to determine whether AQP1 suppresses the p38 MAPK signalling pathway during AKI. The results showed that LPS induced p38 phosphorylation, and AQP1 deficiency significantly increased p38 phosphorylation in LPS-induced RAW264.7 cells at different time points (Fig. 6a–c). Compared with those in the LPS group, the phospho-p38 levels in the siRNA-AQP1 + LPS group at 4, 24 and 48 h after LPS exposure were markedly elevated by 27%, 44%, and 34%, respectively. These observations indicated that AQP1 might reduce the activation of the p38 MAPK pathway in LPS-induced RAW264.7 cells.

### AQP1 protected RAW264.7 cells against LPS-induced inflammation by downregulating the p38 signalling pathway

**Fig. 7:**
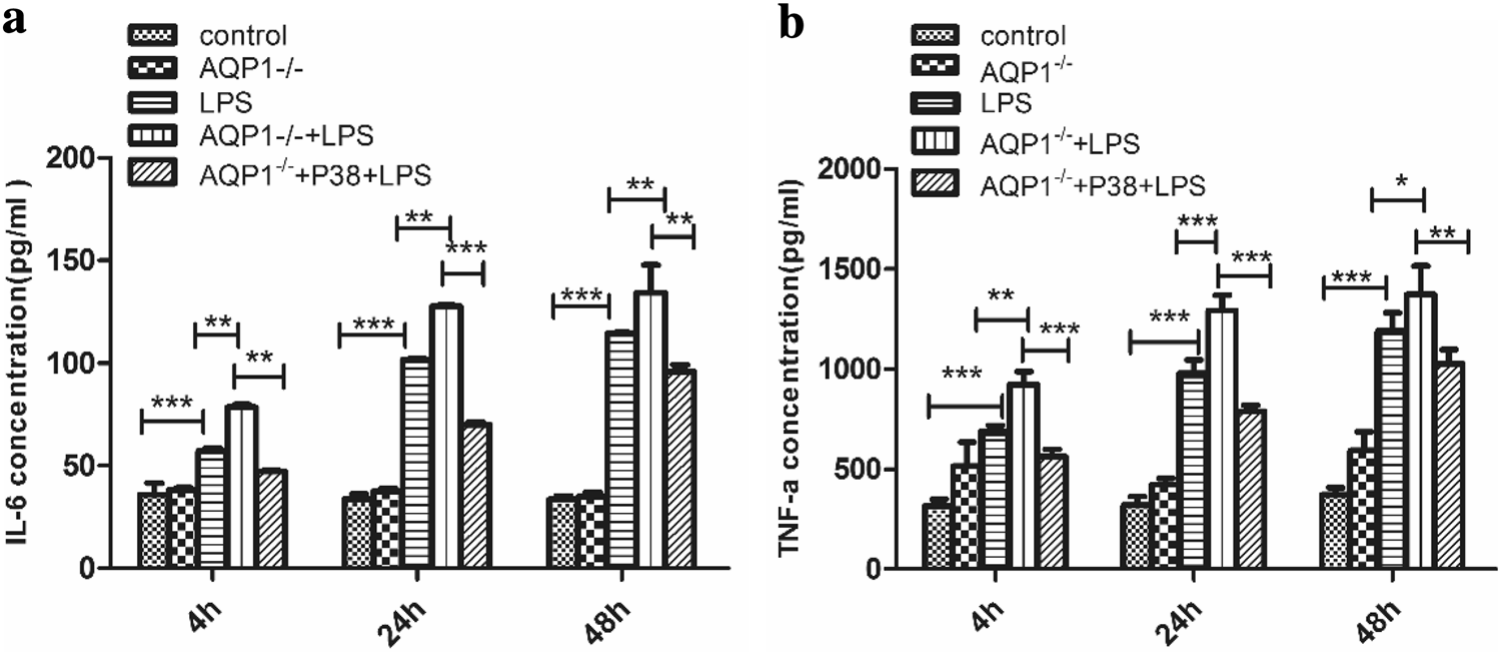
Effects of AQP1 on the production of inflammatory mediators in LPS-induced RAW264.7 cells. **a** The concentration of IL-6 in RAW264.7 cells in different groups was measured by ELISA. **b** The concentration of TNF-α in RAW264.7 cells in different groups was measured by ELISA. The detection time points were 4, 24, and 48 h after LPS stimulation. Student’s *t* test: LPS versus control group; siRNA-AQP1 + LPS versus LPS group; siRNA-AQP1 + Doramapimod + LPS versus siRNA-AQP1 group (*p < 0.05, **p < 0.01, and ***p < 0.001)

In contrast to those in control cells, the IL-6 (Fig. 7a) and TNF-α (Fig. 7b) levels were increased in LPS-treated RAW264.7 cells and increased even more in siRNA-AQP1 + LPS cells. This result proves that silencing AQP1 aggravated the inflammatory reaction. To confirm whether the inhibitory effects of AQP1 on LPS-stimulated inflammatory mediators were due to its influence on p38 MAPK signalling activation, the transfected cells were pretreated with the p38 inhibitor doramapimod (RIRB796) prior to stimulation with LPS as described in the materials and methods. Compared with that in the siRNA-AQP1 + LPS group, changes in inflammatory mediator expression were blocked in the doramapimod-treated group (RIRB796).

### AQP1 inhibited M1 polarisation via suppressing the p38 signalling pathway in LPS-stimulated RAW264.7 cells

**Fig. 8:**
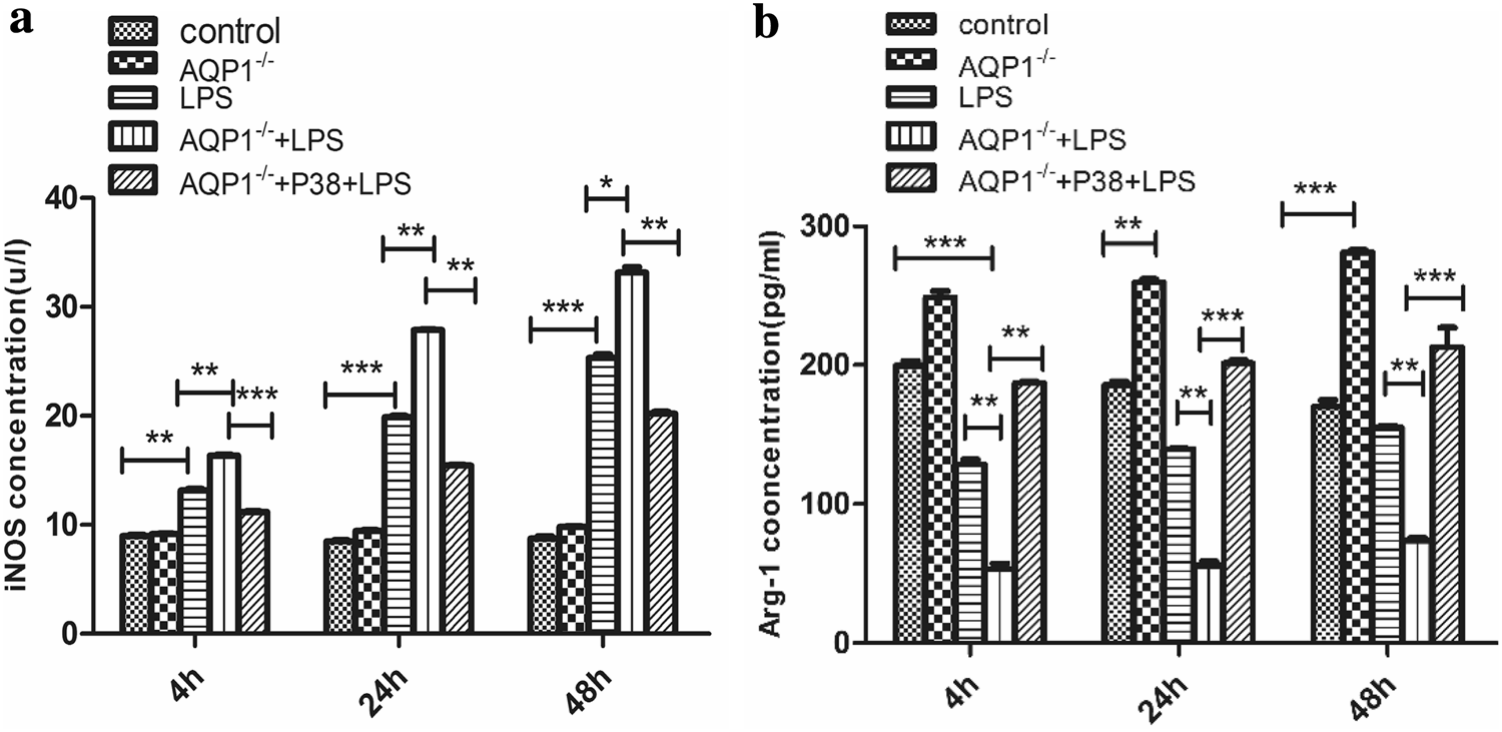
Effects of AQP1 on the phenotypic transition in LPS-induced RAW264.7 cells. The levels of the indicated M1 marker (iNOS) and M2 marker (Arg-1) were quantified by ELISA (**a**, **b**). Student’s *t* test: LPS versus control group; siRNA-AQP1 + LPS versus LPS group; siRNA-AQP1 + Doramapimod + LPS versus siRNA-AQP1 group (*p < 0.05, **p < 0.01, and ***p < 0.001)

In contrast to those in the LPS group, the levels of the M1 marker iNOS were increased in the siRNA-AQP1 + LPS group (Fig. 8a). In contrast, the levels of the M2 marker Arg1 were decreased in the siRNA-AQP1 + LPS group (Fig. 8b). Silencing AQP1 seemed to polarise macrophages towards the M1 phenotype when stimulated by LPS. However, when the cells were pretreated with specific p38 MAPK inhibitors, siRNA-AQP1-mediated increases in iNOS were abolished, indicating that AQP1 might suppress M1 polarisation by inhibiting p38 MAPK activation. The levels of the macrophage markers showed similar trends over time in every group. In addition, compared with those in the other groups, the level of Arg-1 was significantly increased in the siRNA-AQP1 group.

### AQP1 attenuated NF-κB activity via the p38 signalling pathway in LPS-stimulated RAW264.7 cells

**Fig. 9:**
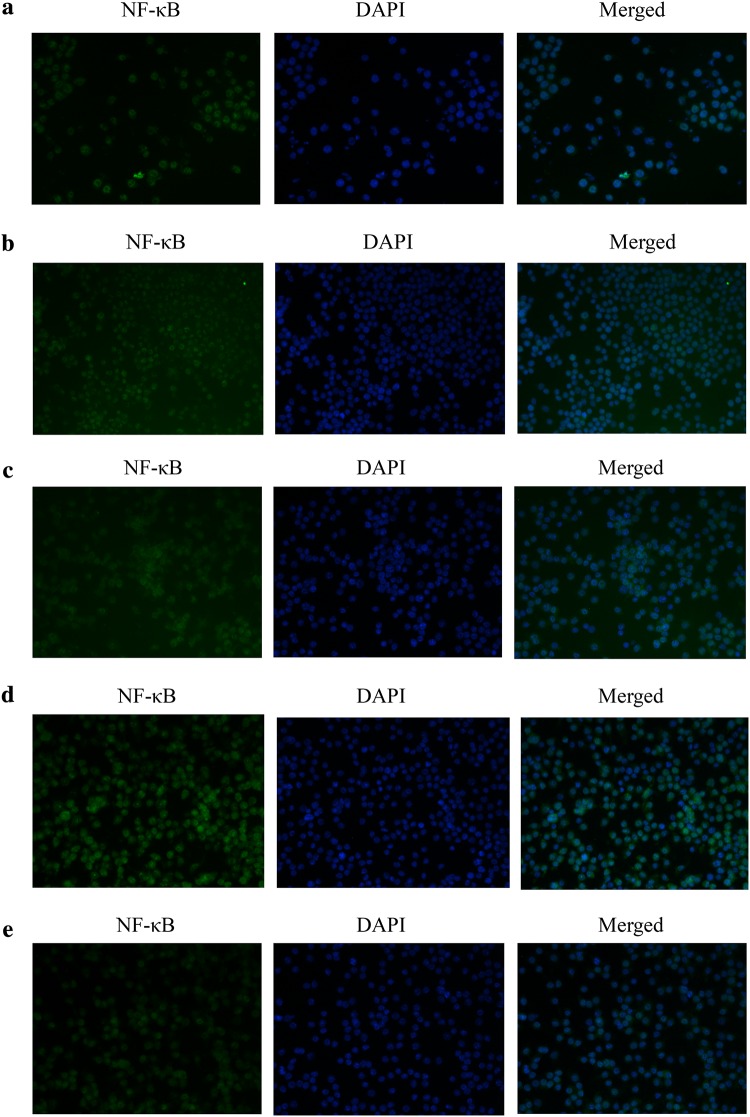
Immunocytochemical localization analysis of NF-κB in RAW264.7 cells. Immunofluorescence assays were conducted using a 24-well Transwell unit. Representative microscopic images of immunofluorescence staining showing the distribution of NF-κB in cells after treatment with 1 μg/ml LPS for 24 h. The data shown are representative of 3 similar observations (the magnification in the images was 400×). **a** Control group. **b** AQP1 group. **c** LPS group. **d** siRNA-AQP1 + LPS group. **e** siRNA-AQP1 + Doramapimod + LPS group

Immunofluorescence staining was used to observe the activation of the nuclear transcription factor NF-κB. Immunofluorescence analysis confirmed that NF-κB was mainly distributed in the cytoplasm in control group cells. LPS stimulation caused NF-κB to translocate from the cytosol to the nucleus, while silencing AQP1 accelerated this translocation (Fig. 9). The activity of NF-κB was reversed in the inhibitor group. These results suggested that the effects of AQP1 on attenuating NF-κB translocation might be related to inhibition of the p38 signalling pathway.

## Discussion

Inflammation is a natural host defence process and leading cause of AKI, which has high morbidity and mortality. Inflammation is largely regulated by macrophages during the innate immune response. Macrophages are known to contribute to inflammation and fibrosis in many kidney diseases [14, 15]. Proinflammatory (classically activated/M1) macrophages are the first responders to injury. After ischaemia/reperfusion acute kidney injury (I/R-AKI), the expression of the M1-specific iNOS was increased within the first 24 h and significantly decreased at 3 days after injury [16]. Upon activation, M1 macrophages were shown to secrete inflammatory cytokines such as IL-1β, TNFα, IL-12, IL-18, and IL-23 [17]. These results were similar to those observed in our study, and we observed changing trends in the expression of these inflammatory factors. M1 macrophage activation and the expression of proinflammatory cytokines were induced at 12 h, peaked at 24 h and then decreased at 48 h after LPS-induced AKI. IL-6 and TNF-α exhibit proinflammatory activity by regulating many intercellular and vascular cell adhesion molecules, causing the recruitment of leukocytes to sites of inflammation [18–20]. iNOS is associated with decreased histological and functional performance during renal injury. In addition, the selective inhibition of iNOS can repair renal microcirculatory derangements caused by sepsis [21]. In our study, the expression of the M2 macrophage marker Arg-1 was obvious at 48 h and 72 h, indicating that renal injury tends to recover at 48 h. The balance between pro- and anti-inflammatory cytokines significantly affects the extent of kidney injury and repair after an acute event. Limiting early macrophage infiltration or activation and protecting against inflammatory factors produced by macrophages represent key approaches that could be used for the prevention or treatment of AKI.

Inflammation is initiated by alterations in cellular and tissue homeostasis. In response to external stimulation, cells normally modulate their internal environment upon the loss or disruption of their ability to regulate fluid movement through the membrane, which leads to the dramatic alteration of cell physiology [22]. The swelling of cells and tissue oedema are typical manifestations of homeostatic disturbance and inflammation. The predominant function of AQPs is the regulation of water transport, and AQPs could be involved in the inflammasome activation regulating cell volume [23]. In vivo experiments revealed that AQP1 mRNA expression was significantly inhibited at 12 h, which was accompanied by significant aggravation of the inflammatory reaction. This result indicated that the development of infection may be associated with the altered expression of AQP1 in AKI. Previous studies have shown that the dysregulation of AQP1 occurs in immune and epithelial cells in response to infectious and inflammatory stimuli. In I/R rats, the urinary exosomal release of AQP1 and AQP2 is reduced in AKI [24, 25], and I/R injury is associated with dramatically reduced expression of AQP1 in the collecting duct and proximal tubule [26]. Our results showed that the decrease in AQP1 occurred earlier than the peak expression of inflammatory factors and that AQP1 expression was a sensitive marker of injury. Notably, the decrease in AQP1 mRNA levels was not accompanied by a parallel decrease in protein expression. The levels of AQP1 in blood and kidney tissues were increased at 12 h and were then significantly decreased at 48 h. The reasons of these inconsistencies may be that: during renal injury, with the decrease of glomerular filtration rate, the excretion of AQP1 in kidney decreased, which increased the accumulation of AQP1 in the systemic circulation and leaded to the increase of AQP1 in blood and kidney tissue. Hiroko Sonoda et al. found the same results; they pointed out that in the early stage of acute kidney injury in rats, the organism retained AQP1 protein in kidney by inhibiting the excretion of AQP1 in urinary exosome [27]. This may be a compensatory protection mechanism of the organism. In addition, a study showed that AQP1 expression is induced in leukocytes in septic patients and is highly expressed during septic shock, demonstrating that AQP1 is involved in regulating the immune response during infection [28]. AQP1 can be considered as an acute phase reactant. During stress, neutrophils, macrophages and other immune cells also synthesise and secrete AQP1 into the blood, which leads to the increase of AQP1 in the early stage of AKI.

To completely understand the role of AQP1 in AKI, we used RNA interference to silence the AQP1 gene in vitro. Silencing AQP1 aggravated the inflammatory reaction and polarised macrophages towards the M1 phenotype. Therefore, we speculated that AQP1 plays a protective role by inhibiting the release of inflammatory factors in LPS-induced AKI. Over many years, studies have focused on the importance of AQP1 in regulating water fluid homeostasis and cell migration, and few researchers have focused on the role of AQP1 in modulating the immune inflammatory response. In mouse vasculitis and intraretinal inflammatory infiltrates, AQP1 protein expression downregulated TNF-α in mouse retinal pigment epithelium (RPE) cells [29]. In a model of acute lung inflammation induced by crystals, AQP1 ablation in macrophages was associated with a marked reduction in IL-1β release and neutrophilic inflammation in the lungs [30]. Liang et al. [31] showed that AQP1 expression can decrease the extent of injury in lung cells by inhibiting TNF-α.

NF-κB is a transcription factor activated by inflammatory responses. In unstimulated cells, NF-κB dimers are present in the cytosol and are bound to the inhibitory IkB protein. When stimulated by proinflammatory stimuli, IκB is rapidly phosphorylated and degraded, followed by the nuclear translocation and DNA binding of NF-κb [32]. In our immunofluorescence staining results, NF-κB was shown to have translocated into the nucleus after LPS administration in both renal tissues and RAW264.7 cells. We also found that AQP1 deletion enhanced the activation of NF-κB, and NF-κB is very important in the pathogenesis of kidney injury. Recent studies have suggested that NF-κB activation in renal tubular epithelial cells exacerbates tubular injury and aggravates inflammation in a kidney I/R model [33].

Previous studies have shown that the MAPK pathway is involved in regulating the responses of inflammatory macrophages towards LPS [34]. The p38 signalling pathway was activated by LPS in our in vitro and in vivo experiments. p38 is the mammalian orthologue of the yeast Hog1p MAP kinase, which was identified in 1994 and isolated as a 38-kDa protein that was rapidly phosphorylated at a tyrosine residue in response to LPS stimulation [35]. Compared with that in LPS-treated cells, the p38 signalling pathway was activated to a greater extent in RAW264.7 cells in which AQP1 was silenced. These results demonstrated that the expression of AQP1 was related to the response of the p38 signalling pathway to inflammation. A recent study led by Umenishi and Schrier demonstrated for the first time that hypertonic-induced AQP1 expression is mediated by activation of the ERK, p38 kinase, and JNK pathways [36]. In staphylococcal peptidoglycan (PGN)-cultured rat pleural mesothelial cells, AQP1 expression was decreased and mediated by activation of the p38 MAPK pathway [37]. Liu and Xie [38] investigated the signalling pathway involved in AQP1 expression caused by LPS in a human pleural mesothelial cell line (MeT-5A). To further elucidate the relationship between AQP1 and the p38 MAPK signalling pathway during inflammatory responses in macrophages, an inhibitor of the p38 pathway, doramapimod (RIRB796), was added to the transfected cells. The expression levels of proinflammatory cytokines and M1 macrophage activation were repressed in the inhibitor group, and the activity of NF-κB was also blocked. These findings indicated that the p38 MAPK signalling pathway was involved in the effects of AQP1 on inflammatory responses in macrophages. AQPs are novel therapeutic targets that modulate oedema, cell migration, and the release of inflammatory cytokines and mediators [39]. p38 plays an essential role in macrophage-mediated inflammation. A recent study reported the protective role of AQP3 in renal injury. AQP3 deletion inhibited the activation of MAPK signalling and significantly increased kidney damage, inflammation and renal oxidative stress [40]. In a sepsis-induced acute renal failure mouse model [41], AQP2 expression was downregulated through the NF-κB pathway, and NF-κB is a downstream transcription factor of the p38 MAPK signalling pathway. In rats with hepatopulmonary syndrome, AQP1 enhanced pulmonary arterial smooth muscle cell (PASMC) migration via the p38 MAPK pathway, which may represent a potential therapeutic strategy for the regulation of pulmonary vascular remodelling [42]. Nevertheless, more experiments are needed to clarify which p38 signalling pathways are involved in the modulation of AQP1 function.

To the best of our knowledge, this is the first study to show that AQP1 expression is related to the inflammatory reaction in macrophages and that the p38 MAPK signalling pathway is involved in the effects of AQP1. In the early stage of AKI, AQP1 mRNA expression was significantly decreased at 12 h after treatment with LPS. However, we did not examine the changes in AQP1 expression before 12 h. In addition, although our findings indicated that AQP1 could promote phenotypic changes, the results did not show a clear linkage between AQP1 and macrophage phenotype transformation in vivo. A previous study reported that AQP1 can promote the migration and proliferation of epithelial cells in proximal tubules and may accelerate the recovery of renal function after acute tubular injury [43]. Functional AQP1 plays a role in repressing polarisation towards the M2 phenotype [44]. The downregulation of AQP1 in macrophages could help to orient macrophages towards tissue remodelling, healing and repair [45]. Macrophages have remarkable heterogeneity in terms of both phenotype and function; therefore, AQP1 in macrophages may have various biological functions in different stages of AKI. In this study, we focused on the relationship between AQP1 and LPS-induced inflammatory responses in macrophages. Our results suggest that AQP1 is a novel therapeutic target for modulating the immune response in inflammatory conditions. Additional investigations should be conducted to understand the mechanism of AQP1 that drives the osmotic stress-induced inflammatory response.

Overall, we demonstrated that AQP1 plays a protective role in modulating acute renal injury. It can attenuate macrophage-mediated inflammatory responses by downregulating p38 MAPK activity in LPS-induced RAW264.7 cells. Our results suggest that AQP1 is an important mediator of AKI and that pharmacological targeting AQP1-mediated p38 MAPK signalling pathways may provide a novel approach for the prevention or treatment of AKI.
